# Apigenin, a dietary flavonoid, inhibits proliferation of human bladder cancer T-24 cells via blocking cell cycle progression and inducing apoptosis

**DOI:** 10.1186/s12935-015-0186-0

**Published:** 2015-03-29

**Authors:** Ming-Der Shi, Cheng-Kai Shiao, Yi-Chieh Lee, Yuan-Wei Shih

**Affiliations:** Department of Medical Technology, Kaohsiung Veterans General Hospital Tainan Branch, Tainan, 71051 Taiwan; Department of Medical Laboratory Science and Biotechnology and Graduate Institute of Biological Technology, Chung Hwa University of Medical Technology, Tainan, 71703 Taiwan; Department of Chest Medicine, Kaohsiung Veterans General Hospital Tainan Branch, Tainan, 71051 Taiwan; Department of Nursing, Chung Hwa University of Medical Technology, Tainan, 71703 Taiwan; Department of Food Nutrition, Chung Hwa University of Medical Technology, Tainan, 71703 Taiwan; Department of Biological Science and Technology and Graduate Institute of Biomedical Science, Chung Hwa University of Medical Technology, Tainan, 71703 Taiwan

**Keywords:** Apigenin, Mitochondrial membrane potential, Cyclin, CDK, Caspase

## Abstract

**Background:**

Apigenin is a nontoxic dietary flavonoid, and it may have chemopreventive and therapeutic potential as an anti-inflammatory, antioxidant, and anti-cancer agent. However, its role in bladder cancer remains poorly understood. The aim of this study was to investigate the anti-proliferative activity of apigenin in human bladder cancer T-24 cells.

**Methods and results:**

Apigenin inhibited T-24 cell proliferation in a dose-dependent manner. We demonstrated that apigenin-induced early and mid-apoptotic cell could be identified by Annexnin V-Alexa Fluor 488/PI apoptosis detection and TUNEL assay. Moreover, using a JC-1 staining assay, we found that apigenin may induce the loss of the mitochondrial membrane potential. By performing flow cytometry and Western blotting, apigenin-mediated subG1 phase acculmulation was also associated with an increase in the phospho-p53, p53, p21, and p27 levels, and with a decrease in the Cyclin A, Cyclin B1, Cyclin E, CDK2, Cdc2, and Cdc25C levels, thereby blocking cell cycle progression. ELISA showed that the subG1 phase acculmulation was due to the increase in the p53, p21, and p27 levels. In addition, apigenin increased the Bax, Bad, and Bak levels, but reduced the Bcl-xL, Bcl-2, and Mcl-1 levels, and subsequently triggered the mitochondrial apoptotic pathway (release of cytochrome *c* and activation of caspase-9, caspase-3, caspase-7, and PARP). Further analysis demonstrated that apigenin increased the ROS levels and depleted GSH in T-24 cells at 12 h.

**Conclusions:**

The results suggested that apigenin inhibits T-24 cells proliferation via blocking cell cycle progression and inducing apoptosis. In addition, we discovered a potential anticancer activity of apigenin against T-24 cells.

## Introduction

Bladder cancer ranks the second most common malignancy of the genitourinary tract and is associated with significant morbidity and mortality [1]. Although most bladder cancer cases are superficial at the time of diagnosis, including surgery, intravesical chemotherapy, radiation/immunotherapy therapy, and system chemotherapy. However, bladder cancer is associated with a poor prognosis, because it is highly resistant to radiation and chemotherapeutic agents. In addition, patients with advanced bladder cancer have a 5-year survival rate of approximately 20-40% despite various treatment modalities [2-4]. Therefore, novel and effective therapeutic strategies for the treatment of advanced bladder cancer are urgently required. Human urinary bladder tumours are known to develop along 2 major and independent biological axes, each presenting with diverse and discrete genetic alterations controlling tumour initiation and progression [5,6]. The complexity of the molecular pathways involved in bladder cancer onset, combined with the various genetic and epigenetic events occurring during tumour progression, are mainly responsible for the profound heterogeneity of this disease [7,8].

Apoptosis plays a major role in the homeostasis and development of tissues in multicellular organisms [9]. Biochemical events lead to cell morphology changes and death. Imbalance between cell proliferation and apoptotic cell death results in critical diseases such as cancer [9,10]. In many cell models, the mode of death produced by an initiation stimulus may be switched by inhibiting control points or major execution steps in the death pathway, such as caspase activation, cytochrome *c* release, or reactive oxygen species (ROS) generation [11-13]. In recent years, scientific interest in mitochondria which plays a vital role in cell death processes. Several stressors, such as inflammation, radiation (ultraviolet or X-rays), heavy metals, drugs, heat shock, and acidification, are inducers of apoptosis, and they are involved in the opening of the permeability transition pore, increase of the Bax/Bcl-2 ratio, and generation of ROS from mitochondria, which may cause the release of apoptogenic factors [14]. Furthermore, intracellular reduced glutathione (GSH) content has a decisive effect on anticancer drug-induced apoptosis, indicating that apoptotic effects are inversely proportional to GSH content [15,16].

Multiple genetic changes occurring during carcinogenesis cause cell abnormalities. Recent advances in cell biology have illustrated the detailed mechanisms of the cell-cycle regulatory systems and have shown that increased cell proliferation is a common characteristic in numerous cancers [17,18]. Cell cycle progression involves a sequential activation of CDKs, the activation of which is dependent on the association with cyclins. Therefore, eukaryotic cells have developed effective and well-regulated mechanisms to control cell-cycle progression [19].

Increased intake of fruits and vegetables has been associated with reduced risks of certain cancers [20]. Apigenin (4′,5,7,-trihydroxyflavone) is a common dietary flavonoid and is widely distributed in several fruits and vegetables, such as parsley, onions, oranges, and tea [21]. Naturally occurring apigenin is found mostly in hydroxylated form, and has been demonstrated to inhibit tumour cell proliferation, motility, angiogenesis, and induce apoptosis [22-25]. Although various studies have shown that apigenin possesses antitumour properties, the mechanisms underlying its antitumour activity remain unknown. In this study, we have employed the human bladder cancer T-24 cell line to understand the molecular mechanisms responsible for the antiproliferative effect of apigenin. We demonstrated that apigenin inhibited T-24 cells proliferation via blocking cell cycle progression and inducing apoptosis.

## Material and methods

### Reagents and antibodies

Apigenin (purity≧99%) was purchased from Extrasynthese (Genay, France); dimethylsulfoxide (DMSO), sodium dodecyl sulphate (SDS), phenylmethylsulfonyl fluoride, and bovine serum albumin (BSA) were purchased from Sigma-Aldrich Chemical Co. (St. Louis, MO, USA); the Annexin V-Alexa Fluor 488 and propidium iodide (PI) apoptosis detection kit were purchased from Invitrogen (Molecular Probe, Inc, Eugene, OR, USA). The protein assay kit was obtained from Bio-Rad Labs. (Hercules, CA, USA). Dulbecco’s phosphate-buffer saline (PBS), and trypsin-EDTA were purchased from Gibco-BRL (Gaithersburg, MD, USA). Mouse- or rabbit-monoclonal antibodies specific for cytochrome c, caspase-3, caspase-7, caspase-9, Cyclin B1, Cyclin E, and CDK2 were purchased from Santa Cruz Biotechnology (Santa Cruz, CA, USA). Mouse- or rabbit-monoclonal antibodies specific for phospho-p53, p53, p21, p27, Bcl-2, Bcl-xL, Mcl-1, Bax, Bad, Bak, poly( ADP-ribose) polymerase (PARP), Cdc2, Cdc25C, and Cyclin A were purchased from Invitrogen Corporation (Camarillo, CA, USA). β-Actin antibody was purchased from BD Transduction Laboratories (San Diego, CA, USA). The enhanced chemiluminescence (ECL) kit was purchased from Amersham Life Science (Amersham, UK).

### Cell culture and apigenin treatment

Human nonmalignant lung fibroblast cell line WI-38 and bladder carcinoma cell line HT-1376 were maintained in MEM medium. Human bladder carcinoma cell line T-24 was maintained in MacCoy’s 5A medium. Human prostate carcinoma cell line PC-3 was maintained in Ham’s F12K medium. The aforementioned cell lines were obtained from BCRC (Bioresource Collection and Research Center, Hsin-Chu, Taiwan). All cells were cultured at 37°C in a humidified atmosphere of 5% CO_2_-95% air. In medium supplemented with 10% fetal calf serum and antibiotics (100 U/ml of penicillin and 100 mg/ml of streptomycin). Adherent cells were detached by incubation with trypsin. For apigenin treatment, the stock solution of apigenin was dissolved in DMSO and sterilised by filtration through 0.2-μm disc filters. Appropriate amounts of the stock solution (1 mg/ml in DMSO) of apigenin were added to the culture medium to achieve the indicated concentrations (final DMSO concentration was < 0.2%).

### Cell viability

To measure the effect of apigenin on cell viability, the WI-38, T-24, HT-1376 and PC-3 cells were seeded in 24-well plates (1 × 10^5^ cells/well) for 16–18 h. The cells were then treated with or without various concentrations (0, 1, 5, 10, 20, 30, 40, and 50 μg/ml) of apigenin for 24 h. Each treatment was repeated 3 times. After the exposure period, the medium was removed and followed by washing the cells with PBS. The medium was then changed and incubated with 3-(4,5-dimethylthiazol-2-yl)-2,5-diphenyltetrazolium bromide (MTT) solution (5 mg/ml)/well for 4 h. The medium was removed, and formazan was solubilised in isopropanol and measured spectrophotometrically at 563 nm. The percentage of viable cells was estimated by comparing them with the untreated control cells.

### Cell-cycle assay

Flow cytometric analysis of T-24 cells was performed using a FACScan flow cytometer (Becton Dickinson Immunocytometry Systems, UK). To analyse cell cycle distribution, the cells were initially treated with various concentrations (0, 1, 5, 10, 20, 30, and 40 μg/ml) of apigenin for 24 h, and were then collected by trypsinisation, fixed in 75% absolute ethanol, washed in PBS, and resuspended in 1 ml of PBS containing 0.5 mg/ml of RNase A and 0.01 mg/ml of PI in the dark for 30 min at room temperature. The cell cycle profiles were analysed using by a flow cytometer. The percentage of cells in the sub-G1, G0/G1, S, and G2/M phases of the cell cycle was analysed using the ModFit LT 3.0 software (Verity Software, Topsham, ME, USA).

### Annexin V-Alexa fluor 488 and PI apoptosis detection assay

Quantitative assessment of apoptosis was performed using the Annexin V-Alexa Flour 488 and PI apoptosis detection kit. Briefly, cells were seeded in 6-well plates, grown for 16–18 h, and treated with apigenin (0, 20, and 30 μg/ml) for the indicated times. After the exposure period, the medium was removed, and the cells were washed with Ca^2+^/Mg^2+^- free PBS. The cells were then fixed with 4% paraformaldehyde in Ca^2+^/Mg^2+^-free PBS for 15 min. In addition, the cells were subsequently washed with annexin-binding buffer [20 mM 4-(2-hydroxyethyl)-1-piperazineethanesulfonic acid (HEPES), 700 mM NaCl, 12.5 mM CaCl_2_, pH 7.4] and stained with Annexin V-Alexa Fluor 488 and PI for 15 min in the dark at room temperature. We then observed the cells under a fluorescence microscope by using a dual filter set for fluorescein isothiocyanate (FITC) and rhodamine (BX51, Olympus, Tokyo, Japan). Apoptotic cells were defined as Annexin V-Alexa Fluor 488-positive and PI-negative cells. Our definition of cellular status is as follows: unstained cells were classified as ‘live’, cells stained for Annexin V-Alexa Fluor 488 only were ‘early apoptotic’, cells stained for both Annexin V-Alexa Fluor 488 and PI were ‘late apoptotic’, and cells stained for PI only were ‘dead’. Apoptotic cells were the sum of early and late apoptotic cells.

### TUNEL assay

We applied a quantitative evaluation method by performing terminal deoxynucleotidyl transferase-mediated deoxyuridine triphosphate nick-end labelling (TUNEL) method to examine DNA strand breaks during apoptosis. In brief, cells were seeded in 6-well plates, and were treated with various concentrations (0, 5, 10, 20, 30, and 40 μg/ml) of apigenin for 6 h. After the exposure period, the medium was removed, and the cells were then washed with Ca^2+^/Mg^2+^- free PBS. Cells were fixed with 4% paraformaldehyde and permeabilised with 0.1% Triton X-100 in 0.1% sodium citrate. After washing, the cells were incubated with the reaction mixture provided in the APO-BrdU^TM^ TUNEL Assay kit (Molecular Probe, Inc). The apoptotic cells with fragmented chromosomal DNA ends were labelled with TdT, which exhibited bead-like green fluorescence under a fluorescence microscope at 20× magnification.

### Mitochondrial-membrane potential assay

We used the mitochondrial-specific cationic dye JC-1 (5,5′,6,6′-tetrachloro-1,1′,3,3′- tetraethyl-benzimidazolylcarbocyanine iodide) (Molecular Probe, Inc), which undergoes potential-dependent accumulation in the mitochondria. JC-1 is selectively accumulated within intact mitochondria to form multimer J-aggregates emitting fluorescence light at 590 nm (red) at a higher membrane potential, and monomeric JC-1 emits fluorescence light at 527 nm (green) at a low membrane potential. The cells were seeded in 6-well plates, and were treated with 0–30 μg/ml of apigenin for 3 and 24 h. After the exposure period, the medium was removed, and the cells were washed with Ca^2+^/Mg^2+^-free PBS. The cells were then stained with 10 μg/ml of JC-1 for 30 min at 37°C and were examined under a fluorescence microscope. Thus, the fluorescence signals were detected by the colour of emitted light by JC-1 indicate the mitochondria membrane potential, which can be analysed by a fluorescence microscope equipped with a dual band-pass filter (detects FITC and rhodamine). Based on the results, we categorised the cells as healthy cells and apoptotic cells. In healthy cells, the dye accumulates and aggregates in the mitochondria, emitting a bright red fluorescence. In apoptotic cells, the dye cannot aggregate in the mitochondria because of the altered mitochondrial membrane potential, and thus it remains in the cytoplasm (monomeric form) and emits a green fluorescence.

### Preparation of whole-cell lysates and Western blotting assay

The cells were lysed with iced-cold radioimmunoprecipitation assay (RIPA) buffer (1% NP-40, 50 mM Tris Base, 0.1% SDS, 0.5% deoxycholic acid, 150 mM NaCl, pH 7.5) and then phenylmethylsulfonyl fluoride (10 mg/ml), leupeptin (17 mg/ml), and sodium orthovanadate (10 mg/ml) were added. After vortexing on ice for 30 min, the samples were centrifuged at 12000 × g for 10 min, and then the supernatants were collected, denatured, and subjected to sodium dodecyl sulphate-polyacrylamide gel electrophoresis (SDS-PAGE) and Western blotting. The protein content was determined using a Bio-Rad protein assay reagent with BSA as a standard. We performed ECL Western blotting was as follows. Proteins were resolved on 10-12% SDS-PAGE gels and then transferred onto nitrocellulose membranes. Nonspecific binding of the membranes was blocked with Tris-buffered saline (TBS) containing 1% (w/v) nonfat dry milk and 0.1% (v/v) Tween-20 (TBST) for more than 2 h. The membranes were washed with TBST 3 times for 10 min and incubated with an appropriate dilution of specific primary antibodies in TBST overnight at 4°C. Subsequently, the membranes were washed with TBST and incubated with an appropriate secondary antibody (horseradish peroxidase-conjugated goat antimouse or antirabbit IgG) for 1 h. After the membranes were washed 3 times for 10 min in TBST, the bands were detected through enhanced chemiluminescence by using ECL Western blotting detection reagents and exposed ECL hyperfilm in FUJIFILM Las-3000 mini-imaging system (Tokyo, Japan). Proteins were then quantitatively determined through densitometry by using FUJIFILM-Multi Gauge V3.0 software.

### Measuring of p53, p21, and p27 levels

Intracellular levels of the levels of p53, p21, and p27 were determined by enzyme-linked immunosorbent assay (ELISA). In brief, T-24 cells were seeded in 5-cm dishes and grown to 85-90% confluence, and were then treated with 0, 20, and 30 μg/ml of apigenin for 6, 12, 24, and 48 h. Cell lysates were placed in 96-well microtiter plates (1 × 10^6^ per well) coated with monoclonal detective antibodies, and then incubated for 2 h at room temperature. After the unbound antibodies were removed through washing with a washing buffer (50 mM Tris, 200 mM NaCl, and 0.2% Tween 20), the detection antibody, which is bound by horseradish peroxidase-conjugated streptavidin, was added to bind to the antibodies. Horseradish peroxidase catalysed the conversion of a chromogenic substrate (tetramethylbenzidine) to a coloured solution with colour intensity proportional to the protein level present in the sample. The absorbance of each well was measured at 450 nm, and p53, p21, and p27 levels were determined by interpolating from standard curves obtained with known levels of standard proteins. Data are expressed as pg p53, p21, or p27 levels (pg/10^6^ cells).

### Measurement of intracellular levels of ROS

Intracellular ROS was estimated using a fluorescent probe, 2′,7′-dichlorofluorescein diacetate (DCFH-DA). DCFH-DA readily diffuses through the cell membrane and is enzymatically hydrolysed by intracellular esterases to non-fluorescent dichlorofluorescin (DCFH), which is then rapidly oxidised to highly fluorescent DCF in the presence of ROS. Briefly, the cells were treated with 30 μg/ml of apigenin for various periods (0, 1, 2, 3, 6, and 12 h). T-24 cells (1 × 10^5^ cells/ml) were then incubated in a culture medium containing 20 μM DCFH-DA for 30 min at 37°C, and were washed with PBS. The cell suspensions were centrifuged at 412 × g for 10 min and the medium was removed. Cells were dissolved with 1% Triton X-100, and DCF fluorescence intensity was detected at various time intervals with an excitation wavelength of 503 nm and an emission wavelength of 529 nm by using the FLUOstar OPTIMA spectrofluorophotometer. DCF fluorescence intensity is proportional to the intracellular ROS levels [26].

### Measurement of intracellular levels of GSH

The intracellular levels of GSH were measured using the method of Hissin and Hilf [27]. After treatment, cells were washed with PBS and scrapped into 6.5% trichloroacetic acid. Phosphate-EDTA buffer (4.5 ml) at pH 8.0 was added to 0.5 ml of the supernatant obtained after centrifugation at 12000 × g. The final assay mixture (2 ml) contained 100 μl of diluted supernatant, 1.8 ml of phosphate-EDTA buffer (pH 8.0), and 100 μl of 0.1% o-phthalaldehyde solution. After thorough mixing and incubation at room temperature for 15 min, fluorescence was read at wavelengths of 350 nm (excitation) and 420 nm (emission) using the FLUOstar OPTIMA spectrofluorophotometer. The reduced form of GSH was used as a standard. Data are expressed as nanomole GSH per 10^6^ cells.

### Statistical analysis

Data are expressed as means ± standard deviation of 3 independent experiments and analysed using Student’s *t*-test (Sigmaplot 2001, SPSS Inc., Chicago, IL, USA). *P* values of <0.05 were considered statistically significant.

## Results

### Apigenin inhibited T-24 cells proliferation

**Figure 1:**
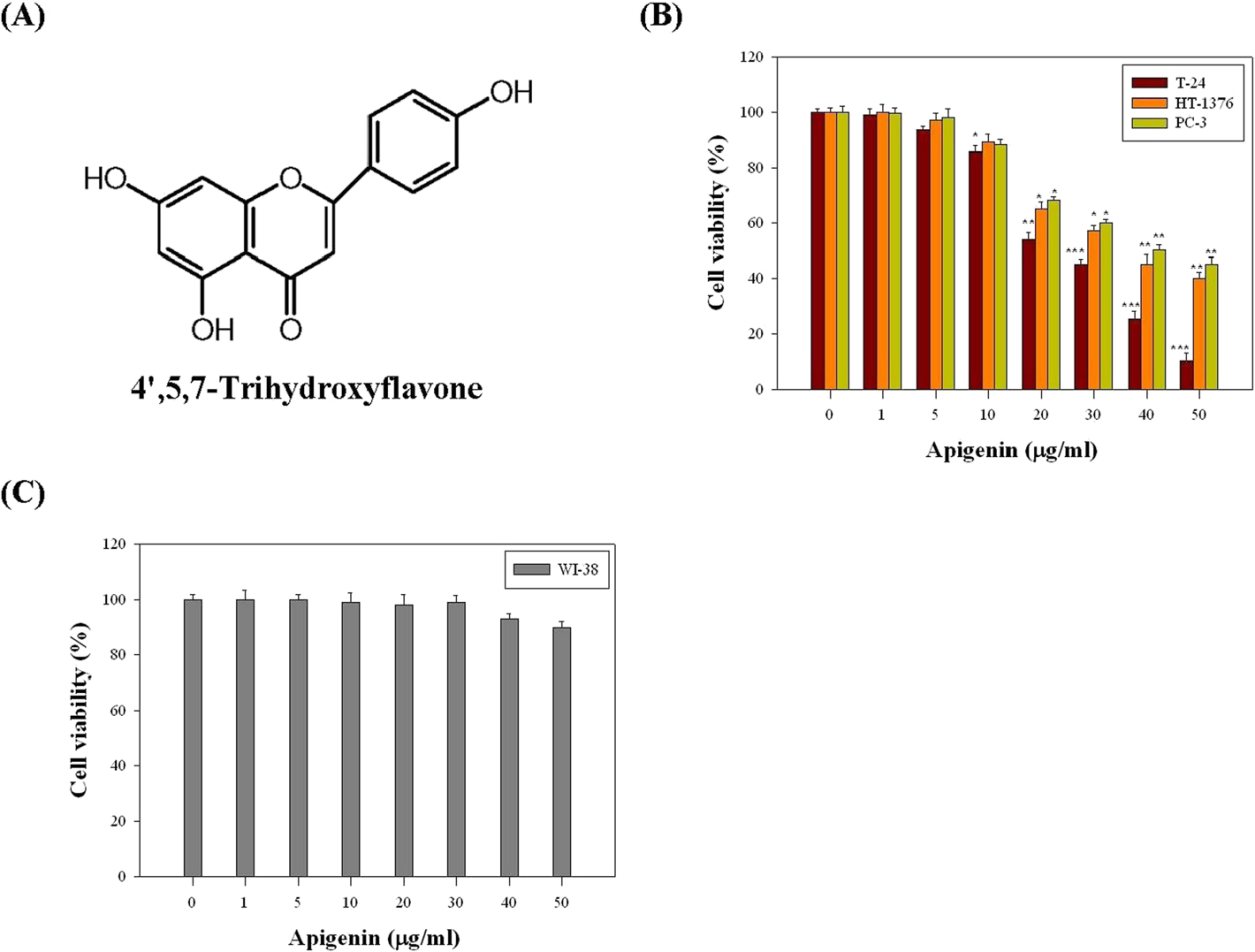
**The effects of apigenin on the viability in four cell lines, WI-38, T-24, HT-1376 and PC-3 cells. (A)** Chemical structure of apigenin. **(B)** Three cultured cells (T-24, HT-1376, PC-3) and **(C)** WI-38 cells were treated with or without apigenin under various concentrations (0, 1, 5, 10, 20, 30, 40, and 50 μg/ml) for 24 h. Thereafter, cell viabilities were determined by MTT assay. The survival cell number was directly proportional to formazan, which was measured spectrophotometrically at 563 nm. Values represent mean ± SD of three independent experiments (*P < 0.05, **P < 0.01, ***P < 0.001 compared with the untreated control (dose 0).

The chemical structure of apigenin is illustrated in Figure 1A. To investigate the effect of apigenin on cell viability in the four cell lines (WI-38, T-24, HT-1376 and PC-3), the cell viability was determined using a MTT assay. The results showed that apigenin reduced the viability of these three cell lines, T-24, HT-1376, and PC-3 in a dose-dependent manner (Figure 1B). Particularity, the IC_50_ values of apigenin were 23.6, 35.2, and 40.2 μg/ml for T-24, HT-1376, and PC-3 cells at 24 h, respectively. Among these cells, T-24 cells were much more sensitive to apigenin compared with the other cell lines. Furthermore, the strongest potency of apigenin on the cytotoxicity of cancerous cells was toward T-24 bladder cancer cells; therefore, we selected the T-24 cell line for subsequent experiments. Apigenin had no cytotoxic effect towardsWI-38 cells (Figure 1C).

### Apigenin induced cell cycle arrest and apoptosis in T-24 cells

**Figure 2:**
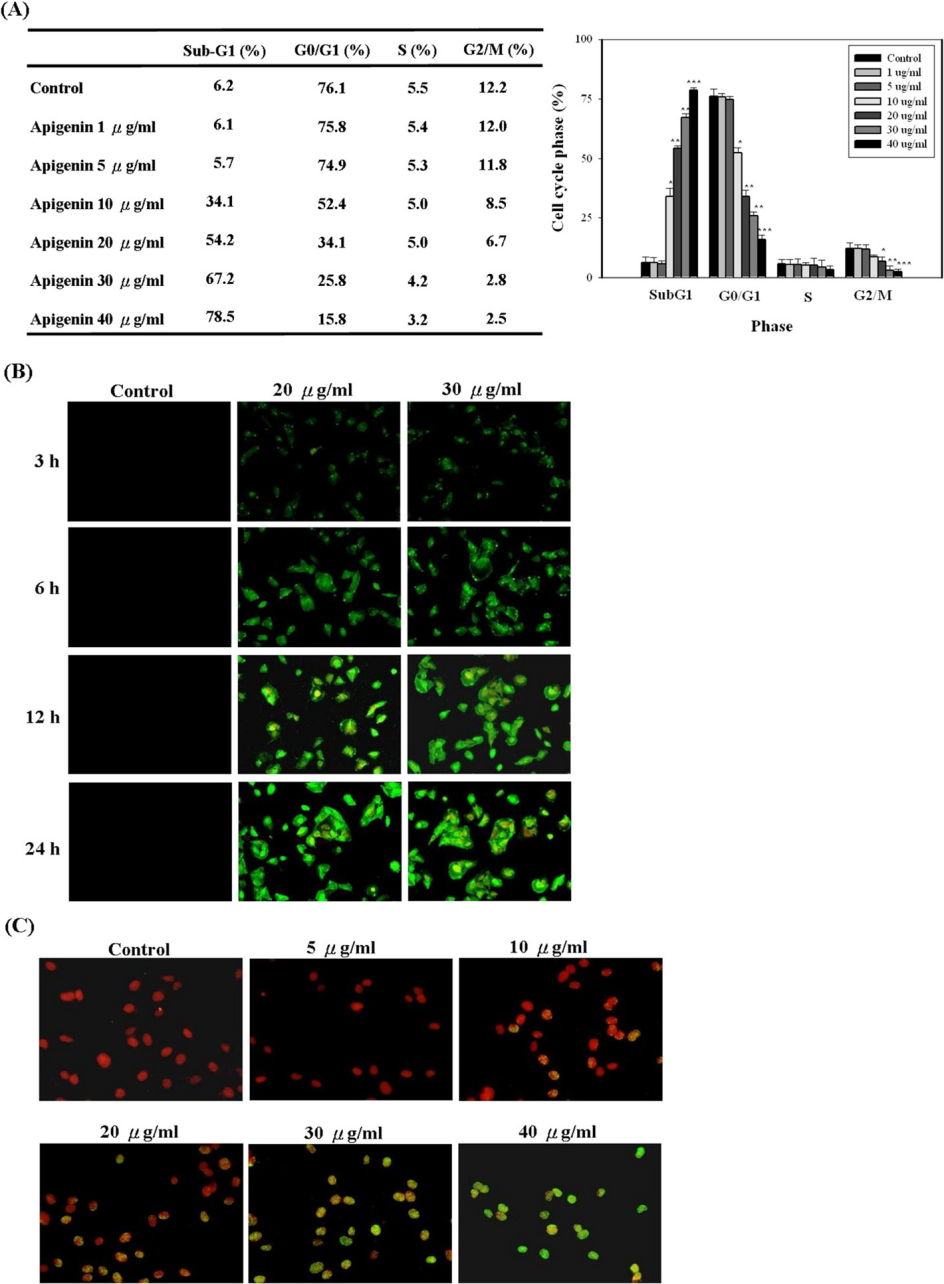
**Apigenin inhibited cell cycle progress and induced apoptosis in T24 cells. (A)** Cultured cells were treated with or without apigenin under various concentrations (0–40 μg/ml). Twenty-four hours later, the cell cycle distribution was analysed by flow cytometry. The data indicate the percentage of cells in sub-G1, G0/G1, S, and G2/M phases of the cell cycle. **(B)** Cells were treated with apigenin (0, 20, and 30 μg/ml) for the indicated times, and then the induction of apoptosis was assessed by Annexin V-Alexa Fluor 488/PI assay kit. **(C)** Cells were treated with or without apigenin under various concentrations (0–40 μg/ml), and then the induction of apoptosis was assessed by TUNEL assay kit. The results represented the average of three independent experiments ± S.D. *P < 0.05, **P < 0.01, ***P < 0.001 compared with the untreated control.

To test the underlying mechanism that leads to the apigenin-induced loss of cell proliferation, we observed the effects of apigenin on T-24 cells by detecting the apoptotic effect and cell cycle progression. Briefly, T-24 cells were treated with 0, 1, 5, 10, 20, 30, and 40 μg/ml of apigenin for 24 h and subjected to flow cytometry. The cells were treated with apigenin (0–40 μg/ml) for 24 h, an apparent accumulation of cells in the sub-G1 phase (at the hypodiploid phase, also named subG1 phase) from 6.2% to 78.5% was observed (Figure 2A). The subG1 phase increased (greater than 50%) when the cells were treated with 20–40 μg/ml of apigenin. In addition, apigenin treatment significantly reduced the percentage of cells in the G2/M phase. No change in the S phase was observed. Thus, these results showed that apigenin inhibited T-24 cells proliferation.

Subsequently, we assessed the effects of apigenin on the induction of apoptosis in T-24 cells by performing Annexin V-Alexa Fluor 488/PI assay and TUNEL assays. First, the Annexin V-Alexa Fluor 488/PI double-staining technique was performed to investigate whether apigenin induced early apoptosis in T-24 cells. The percentage of T-24 cells undergoing early apoptotic cell death was increased by apigenin in a dose- and time-dependent manner (Figure 2B). To further confirm apigenin-induced apoptosis, a TUNEL assay was performed to detect DNA-strand breaks. The assay results revealed that exposure of T-24 cells to 5–40 μg/ml of apigenin for 6 h, resulted in an appreciable increase of fluorescein-stained nuclei compared with untreated control cells (Figure 2C).

### Apigenin-induced the loss of mitochondrial membrane potential and the release of cytochrome *c* in T-24 cells

**Figure 3:**
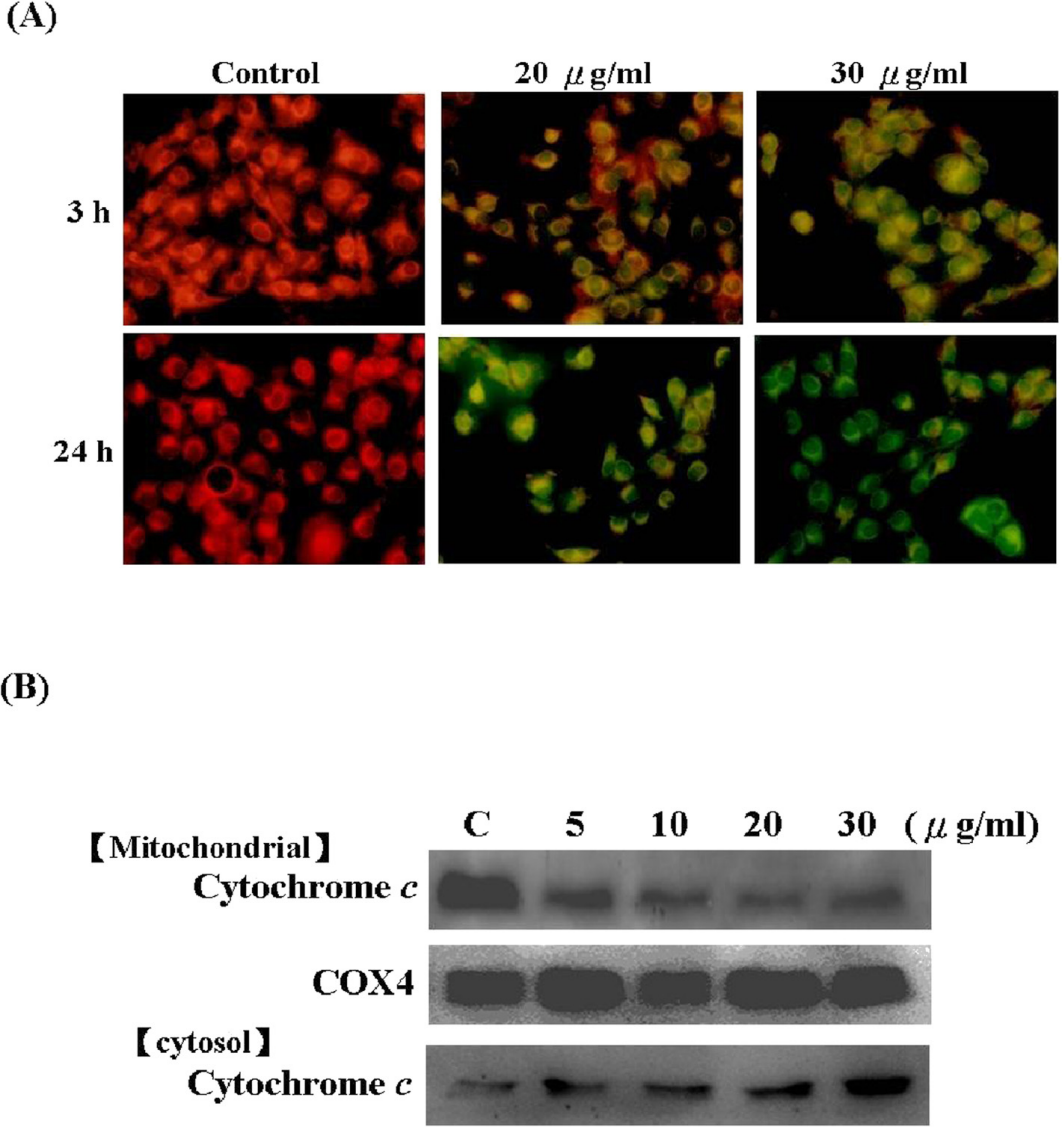
**Apigenin induced the loss of mitochondrial membrane potential and the release of cytochrome** ***c*** **in T-24 Cells. (A)** Cultured cells were treated with indicated concentration (0, 20, and 30 μg/ml) of apigenin for 3 and 24 h. And then, mitochondrial membrane potential was measured by fluorescence microscope using JC-1 staining. **(B)** Cultured cells were treated with indicated concentration (0, 5, 10, 20, and 30 μg/ml) of apigenin for 24 h, and the expressions of cytochrome *c* in cytosol and mitochondria were assayed by Western blotting. The results were represented by using an ECL system and COX-4 was the internal control. The folds were compared with control. The results were obtained from at least three independent measurements.

The loss of mitochondrial membrane potential is an early event in apoptosis. Therefore, we assessed the effect of apigenin treatment on the mitochondrial membrane potential (△Ψ*m*) by using a fluorescence microscope and JC-1 fluorescent dye. As shown in Figure 3A, When the T-24 cells were incubated with 0–40 μg/ml of apigenin for 3 and 24 h, the intensity of red fluorescence decreased, whereas that of green fluorescence increased in the cytoplasm with an increase in the apigenin level. Further indication of the loss of mitochondrial membrane potential were more obvious in high-dose (20 and 30 μg/ml) apigenin-treated cells, By contrast, when the T-24 cells were treated with 5 and 10 μg/ml of apigenin for 3 and 24 h, no change was observed in the mitochondrial membrane potential (data not shown). Moreover, T-24 cells were incubated with 20 and 30 μg/ml of apigenin for 24 h drastically reduced the mitochondrial membrane potential by approximately 50% and 85% respectively. Disruption of the mitochondrial membrane results in the release of cytochrome *c* from the mitochondrial into the cytosol; consequently, cytochrome *c* can be detected by Western blotting. Our results revealed that apigenin treatment of T-24 cells led to the release of cytochrome *c* from the mitochondria in a dose-dependent manner (Figure 3B).

### Apigenin induced apoptotic death through the mitochondrial apoptotic pathway in T-24 cells

**Figure 4:**
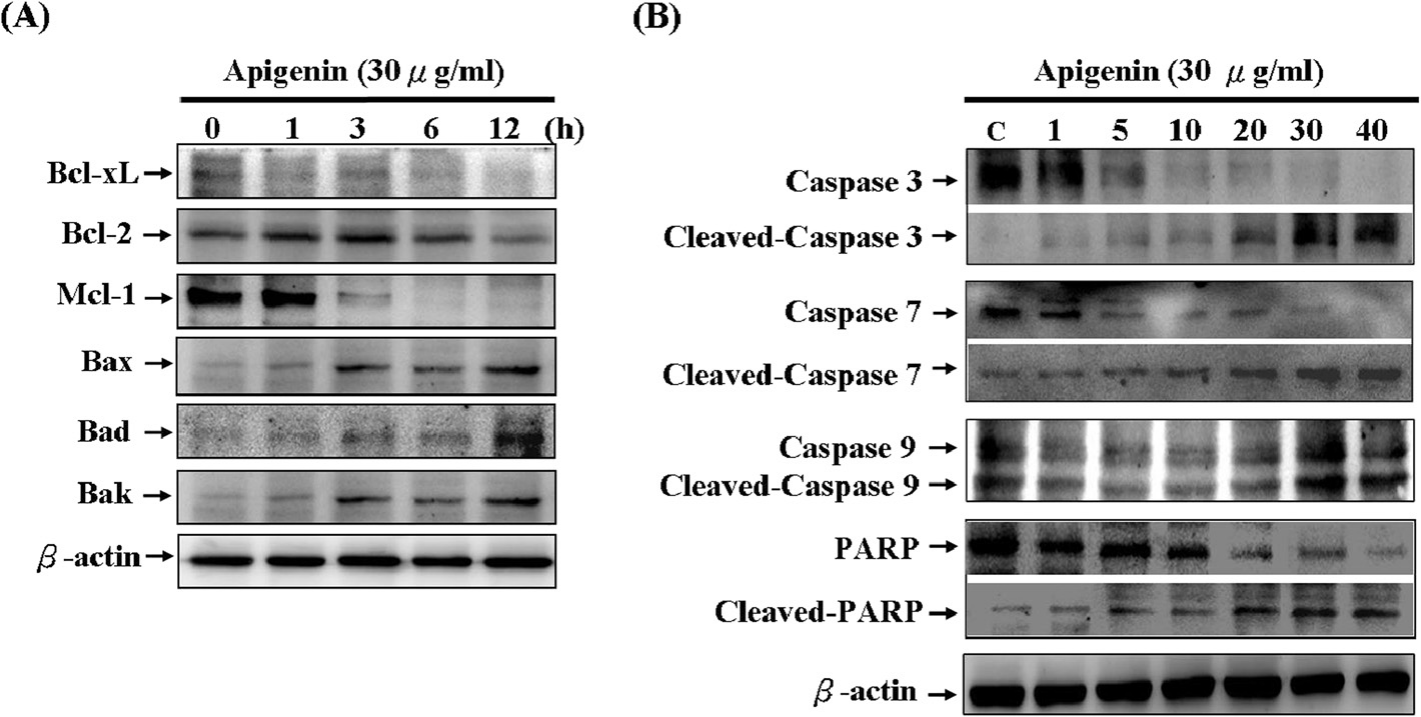
**Apigenin on the protein levels of proapoptotic proteins, antiapoptotic proteins, and the activation of the caspases cascade in T-24 cells.** Cell were treated with various concentrations of apigenin for 24 h and 30 μg/ml apigenin for various periods of time (0, 1, 3, 6, and 12 h), and total cell lysates were prepared to detect **(A)** the protein levels of Bcl-xL, Bcl-2, Mcl-1, Bax, Bad, and Bak. **(B)** cleavage of caspase-3, caspase-7, caspase-9, and PARP by Western blotting analysis. The results were represented by using an ECL system and β-actin was the internal control. The folds were compared with control. The results were obtained from at least three independent measurements.

We investigated whether apigenin suppresses the viability of T-24 cells and promotes DNA fragmentation in such cells through the activation of the mitochondrial (intrinsic) apoptotic pathway. To determine the mitochondrial apoptotic events involved in apigenin-induced apoptosis, we first analysed the changes in the levels of proapoptotic proteins Bax, Bad, and Bak, and antiapoptotic proteins Bcl-xL, Bcl-2, and Mcl-1. The results showed that apigenin treatment of T-24 cells increased the Bax, Bad, and Bak protein levels. By contrast, apigenin decreased the Bcl-xL, Bcl-2, and Mcl-1 protein levels (Figure 4A).

The activation of cysteine proteases, which are both initiators and executors of cell death, is the hallmark of apoptosis [28]. The results indicated that apigenin significantly activated caspase-3, caspase-7, and caspase-9 and induced marked cleavage of PARP in T24 cells (Figure 4B).

### Effects of apigenin on the expression of cell cycle-related proteins

**Figure 5:**
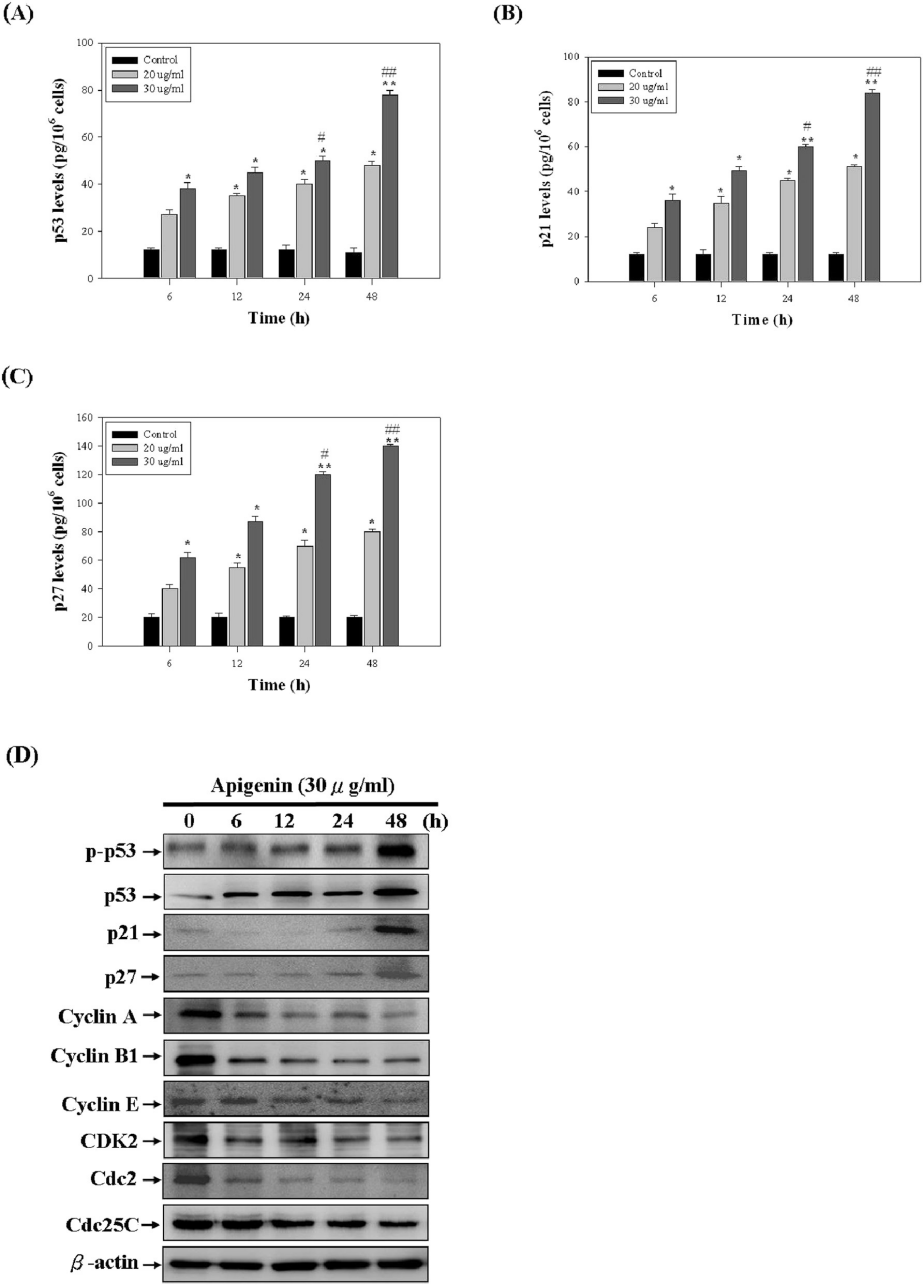
**The effect of apigenin on cell cycle-related molecules in T-24 cells. (A, B, C)** The levels of p53, p21, and p27 in T-24 cells were measured by ELISA kit. **(D)** The levels of p-p53, p53, p21, p27, Cyclin A, Cyclin B1, Cyclin E, CDK2, Cdc2, and Cdc25C were assessed by Western blotting assay. The results represented the average of three independent experiments ± S.D. *P < 0.05, **P < 0.01 compared with the untreated control; #*P <* 0.05, ##*P <*0.01 compared with the 0 h-treated time.

To identify the pathway involved in apigenin-induced cell cycle arrest in T-24 cells, a series of experiments were investigated in which the effects of apigenin on cell cycle regulatory molecules. First, we measured the protein expression and phosphorylation status of p53 by using ELISA and Western blotting. The protein expression and phosphorylation status of p53 increased significantly in a time-dependent manner after treatment with 30 μg/ml of apigenin (Figure 5A and D). Furthermore, the CDK inhibitors p21 and p27 were involved in cell cycle arrest. Thus, we examined the effects of apigenin on the p21 and p27 protein levels. The results unexpectedly indicated increases in the p21 and p27 protein levels at 24 h reaching a maximum at 48 h in a time-dependent manner (Figure 5B and C). We concluded that apigenin-induced cell cycle arrest was associated with an induction in the p53 protein level and p53 phosphoryaltion that subsequently modulated the p21 and p27 protein levels. In addition, we also assessed the effects of apigenin treatment on the Cyclin A, Cyclin B1, Cyclin E, CDK2, Cdc2, and Cdc25C protein levels, which are regulators of cell cycles. Our results indicated that treatment with 30 μg/ml of apigenin reduced the Cyclin A, Cyclin B1, Cyclin E, CDK2, Cdc2, and Cdc25C protein levels in a time-dependent manner in T24 cells (Figure 5D).

### Effects of apigenin on the intracellular levels of ROS and GSH in T-24 cells

**Figure 6:**
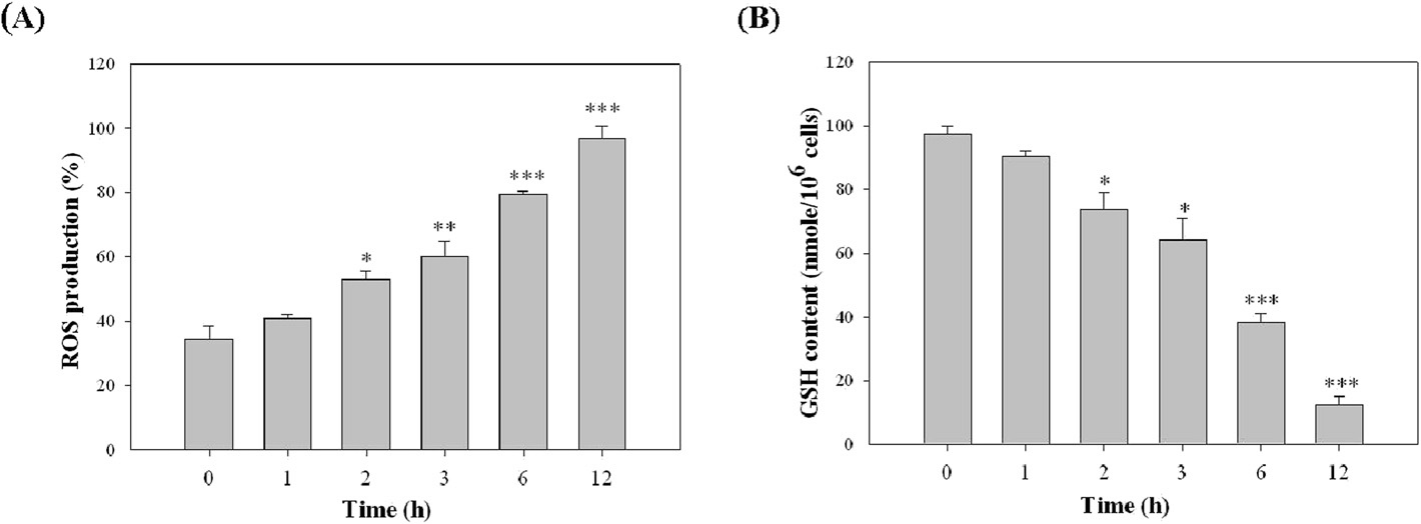
**The effect of apigenin on the intracellular levels of ROS and GSH in T-24 cells.** Cells were treated with 30 μg/ml apigenin for various periods (0, 1, 2, 3, 6, and 12 h), and then were analysed for **(A)** the intracellular ROS production **(B)** the intracellular GSH content by spectrofluorophotometer as described in “Materials and Methods” section. The results represented the average of three independent experiments ± S.D. *P < 0.05, **P < 0.01, ***P < 0.001 compared with the 0 h-treated time.

Several reports have suggested that ROS act upstream of caspase-3 activation in signal transduction pathways, and thus lead to apoptosis. Furthermore, oxidative reactions in the mitochondria result in the ROS generation, which are converted to H_2_O_2_ by superoxide dismutase. The nonenzymatic glutathione (γ-glu-cys-gly) is the most effective antioxidant that prevents ROS generation. Depletion of GSH is associated with the apoptotic cell death machinery [29]. As shown in Figure 6A, apigenin induced an increase in intracellular ROS in a time-dependent manner. One of the consequences of ROS generation is scavenging of free radicals by GSH. The intracellular levels of GSH were measured in apigenin-treated T-24 cells at various time points. The GSH levels were time-dependently decreased in apigenin- treated T-24 cells (Figure 6B).

## Discussion

Bladder cancer is the second most common cancer of the genitourinary tract. Recent research has identified the food compounds (phytochemicals) that may have crucial anticarcinogenic activities. Chemopreventive phytochemicals can suppress and delay the initiation or reverse the promotion stage in multistep carcinogenesis. They can block cancer progression by various mechanisms, including acting as antiproliferative agents or antioxidants. Certain fruits and vegetables possess diverse pharmacological properties and are rich sources of phytochemicals with anticarcinogenic potential [30,31].

Flavonoids are bioactive phytochemicals that are widely distributed in plants and are capable of being absorbed without preceding hydrolysis by the gastrointestinal tract. They are ubiquitously found in fruits, vegetables, tea, and wine and as plant secondary metabolites [32-34]. During the last decade, numerous studies have shown that flavonoids and their metabolites have various pharmacological properties. Flavonoids have a backbone of 2-phenylchromen-4-one (2-phenyl-1-benzopyran-4-one). Recent study has demonstrated that apigenin could enhance anticarcinogenic effects against ultraviolet B (UVB)- and benzo(a)pyrene (BaP)-induced skin tumour and mitochondrial dysfunction in mice. Data on skin tumorigenesis in mice have clearly shown that pretreatment with apigenin significantly suppresses UVB-induced skin tumour incidence [35]. Therefore, apigenin is a glycoside form of flavonoid which has physiological benefits when consumed in its natural food form. Cell viability was assayed in cultures exposed to 0–50 μM apigenin for 24 h, and apigenin exhibited a dose-dependent inhibitory effect on the growth of T-24, HT-1376, and PC-3 cells. Compared with the other cancer cell lines, apigenin had a more effective inhibitory effect on the growth of T-24 (human bladder carcinoma cells). However, WI-38 (human nonmalignant lung fibroblast cells) was less sensitive to the inhibitory growth effect of apigenin than the other cancer cells, indicating that tumour cells are more responsive to the apigenin treatment. One of the major criteria for potential anticancer drugs is the ability to selectively kill tumour cells but not normal cells. Furthermore, our present data have revealed apigenin treatment resulted in a dose-dependent accumulation of T-24 cells in the sub G1 phase concomitantly with a reduction in the G2/M phase. No change in S phase was observed. Our observations are similar to those of a study on piceatannol, a natural polyphenol present in grapes and wine. In that study, piceatannol diminished the accumulation of human T-24 and HT1376 bladder cancer cells in the G2/M phase of the cell cycle [36].

Further investigations are required on the association between arrest of the G1 phase and p-p53, p53, p27, and p21 and on the effect of apigenin on cyclins, CDKs, Cdc2, and Cdc25C, which is associated with cyclins and CDKs that regulate cell cycle progression in response to apigenin treatment. Tumour suppressor gene p53 is well-known to play a crucial role in inducing apoptosis and cell cycle arrest after DNA damage or cellular stress in human cells [37]. Furthermore, p53 is regarded as acting as a cellular gatekeeper for growth and division by controlling critical cell cycle checkpoints. p53 mediates apoptosis by activating APO-1/Fas and other death receptors by upregulating and downregulating Bax and Bcl-2, respectively. p53 is also involved in mitochondrial generation of ROS from mitochondria, which may cause the release of apoptotic factors. p21 and p27 proteins inhibit the activities of various cyclin-dependent kinases, thereby blocking the G1 to S phase transition in the cell cycle [38]. Previous studies showed that p21 and p27 are transcriptionally regulated by p53-dependent and p53-independent pathways [39]. Our results showed that apigenin treatment of T-24 cells results in an increase in the p-p53, p53, p21, and p27 levels. Therefore, we suggest that the blockade of cell cycle progression was caused by the increase in the p21 and p27 protein levels, thereby inhibiting T-24 cells proliferation. Eukaryotic cell cycle progression involves a sequential activation of CDKs, whose activation is dependent upon their association with cyclins. In our study, we demonstrated that apigenin decreased the Cyclin A, Cyclin B1, Cyclin E, CDK2, Cdc2, and Cdc25C protein levels, whereas it increased the p21, p27, p53, and p-p53 protein levels.

The mitochondrial apoptotic pathway has been considered a major signalling pathway of apoptotic cell death in mammalian cells. The mitochondrial membrane potential (△Ψ*m*) often decreases in apoptosis. This decrease in △Ψ*m* is mediated by the opening of permeability transition pores [40]. In addition, electron transport and oxidative phosphorylation are disrupted and ROS frequently accumulate during apoptosis, suggesting a dysfunction of mitochondria. Generated ROS include the superoxide anion radical (.O_2_^−^), hydroxyl radical (.OH), hydrogen peroxide (H_2_O_2_), and peroxynitrite (ONOO^−^) [41]. The generation of ROS and the resulting oxidative stress are implicated as cell death initiation signals that contribute to mitochondrial dysfunction (e.g. a decrease in △Ψ*m*). The nonenzymatic glutathione (c-glu-cys-gly) is the most effective antioxidant that prevents ROS generation. Depletion of GSH is associated with the apoptotic cell death machinery, because a decrease in the GSH levels and concomitant increase in ROS during apoptosis has been reported [29]. Our study demonstrated that apigenin blocked cell cycle progression and induced late cell death in cancer cells via an intrinsic pathway to reduce the GSH levels through the excessive accumulation of intracellular ROS.

Furthermore, mitochondrial function is regulated by the members of the Bcl-2 family, comprising antiapoptotic proteins (Bcl-2, Bcl-XL, and Mcl-1) and proapoptotic proteins (Bax, Bad, and Bak), which control the permeability of the mitochondrial membrane and play a major role in the intrinsic apoptotic pathway [42]. After apigenin treatment of T-24 cells, we observed a significant increase in the expression of Bax, Bad, and Bak, and a decrease in the expression of Bcl-2, Bcl-XL, and Mcl-1. These findings suggest that changes in the ratio of the proapoptotic and antiapoptotic proteins of the Bcl-2 family might contribute to the apoptosis-promoting activity of apigenin. In addition, our results indicated that apigenin treatment of T-24 cells reduces △Ψ*m* and activates caspase-9, caspase-3, and caspase-7. Furthermore, caspase-3 activation leads to cleavage of numberous proteins, including PARP. Cleavage of PARP is a major indicator of apoptosis. This polymerase is a nuclear DNA-binding zinc finger protein that plays a role in DNA repair and in other cellular processes, including cell proliferation, differentiation, and apoptosis [43]. Consistent with this finding, apigenin-induced apoptosis, which involves mitochondrial apoptotic events, was associated with the modulation of the Bcl-2 family of proteins by apigenin. These results confirm the involvement of caspases in apoptotic response to apigenin in T-24 cells, and this involvement was confirmed by immunological detection of the 116 kDa intact PARP and appearance of its 85 kDa fragment, corresponding to the product released on caspase activation.

In summary, the present study results indicated that (1) human bladder cancer T-24 cells are highly sensitive to growth inhibition by apigenin; (2) apigenin can block cell cycle progression in the subG1 phase, thereby inhibiting T-24 cells proliferation; (3) apigenin can inhibit cell cycle progression involving p53 upregulation, increase the expressions of p21 and p27, and reduce the expression of Cyclin A, Cyclin B1, Cyclin E, CDK2, Cdc2, and Cdc25C; (4) apigenin triggers the mitochondrial apoptotic pathway by regulating the expression of Bcl-2 family proteins, causing the release of cytochrome *c*, and activating caspase-9, caspase-7, caspase-3, and PARP. These findings suggest that apigenin may be an effective chemopreventive agent for bladder cancer.
